# The Multiple Roles of Ascorbate in the Abiotic Stress Response of Plants: Antioxidant, Cofactor, and Regulator

**DOI:** 10.3389/fpls.2021.598173

**Published:** 2021-04-12

**Authors:** Minggang Xiao, Zixuan Li, Li Zhu, Jiayi Wang, Bo Zhang, Fuyu Zheng, Beiping Zhao, Haiwen Zhang, Yujie Wang, Zhijin Zhang

**Affiliations:** ^1^Biotechnology Research Institute, Heilongjiang Academy of Agricultural Sciences, Harbin, China; ^2^Biotechnology Research Institute, Chinese Academy of Agricultural Sciences, Beijing, China; ^3^National Key Facility of Crop Gene Resources and Genetic Improvement, Beijing, China

**Keywords:** ascorbate, abiotic stress, antioxidant, redox signal, phytohormone

## Abstract

Ascorbate (ASC) plays a critical role in plant stress response. The antioxidant role of ASC has been well-studied, but there are still several confusing questions about the function of ASC in plant abiotic stress response. ASC can scavenge reactive oxygen species (ROS) and should be helpful for plant stress tolerance. But in some cases, increasing ASC content impairs plant abiotic stress tolerance, whereas, inhibiting ASC synthesis or regeneration enhances plant stress tolerance. This confusing phenomenon indicates that ASC may have multiple roles in plant abiotic stress response not just as an antioxidant, though many studies more or less ignored other roles of ASC in plant. In fact, ACS also can act as the cofactor of some enzymes, which are involved in the synthesis, metabolism, and modification of a variety of substances, which has important effects on plant stress response. In addition, ASC can monitor and effectively regulate cell redox status. Therefore, we believe that ASC has atleast triple roles in plant abiotic stress response: as the antioxidant to scavenge accumulated ROS, as the cofactor to involve in plant metabolism, or as the regulator to coordinate the actions of various signal pathways under abiotic stress. The role of ASC in plant abiotic stress response is important and complex. The detail role of ASC in plant abiotic stress response should be analyzed according to specific physiological process in specific organ. In this review, we discuss the versatile roles of ASC in the response of plants to abiotic stresses.

## Introduction

Ascorbate (ASC, known as vitamin C) is an essential micronutrient for humans, and its deficiency can cause several serious diseases, such as scurvy (Baron, 2009; Carpenter, 2012). In plants, it also participates in many physiological processes, such as photosynthesis, cell division, and differentiation, and is crucial for plant growth and development and adaptation to stress (Nickle and Meinke, 1998; de Pinto et al., 1999; de Pinto and De Gara, 2004; Sun et al., 2010; Gallie, 2013; Kromdijk et al., 2016; Akram et al., 2017; Foyer et al., 2020).

Ascorbate has a critical role in scavenging reactive oxygen species (ROS) in plants under abiotic stresses, such as high-intensity light, high salinity, and drought. These stresses can cause ROS accumulation in plants, which severely damages cell composition and disturbs growth and development (Schieber and Chandel, 2014; You and Chan, 2015; Choudhury et al., 2017). As an antioxidant, ASC can effectively scavenge the accumulated ROS *via* direct or indirect pathways, and is thus critical for eliminating oxidative damage and enhancing abiotic stress tolerance in plants (Noctor and Foyer, 1998; Akram et al., 2017; Hasanuzzaman et al., 2019; Broad et al., 2020a; Elkelish et al., 2020).

In addition to being an antioxidant, ASC can act as a cofactor of certain oxidases, such as the 2-oxoglutarate-dependent dioxygenases (2-ODDs), and participates in the biosynthesis of several phytohormones (Prescott and John, 1996; Arrigoni and De Tullio, 2002; Brisson et al., 2012; Terzi et al., 2015; Mir et al., 2018; Bilska et al., 2019). For example, it is a cofactor of the aminocyclopropane-1-carboxylic acid oxidases (ACOs) and 9-*cis*-epoxycarotenoid dioxygenases (NCEDs), key enzymes in the biosynthesis of the phytohormones ethylene and abscisic acid (ABA), respectively (Qin and Zeevaart, 1999; Brisson et al., 2012; Houben and Van de Poel, 2019). Phytohormones play important roles in regulating plant responses to abiotic stresses. Hence, ASC participates in plant abiotic stress responses through phytohormone pathways (Chen et al., 2014; Terzi et al., 2015; Bilska et al., 2019; Foyer et al., 2020). Further, as a cofactor, it participates in epigenetic modification, and it regulates plant abiotic stress responses *via* epigenetic pathways (Chowrasia et al., 2018; Song et al., 2018).

Moreover, ASC has an important effect in cell signaling (Pignocchi and Foyer, 2003; Chen and Gallie, 2004; Foyer et al., 2020). It plays a critical role in maintaining plant extracellular and intracellular redox homeostasis (Noctor and Foyer, 1998; Ding et al., 2020). Plant redox homeostasis is involved in stress signal transmission, and has a profound effect on multiple signaling pathways, such as the ROS, ABA, and auxin signaling pathways (Noctor and Foyer, 1998; Arrigoni and De Tullio, 2002; Pignocchi et al., 2006; Lima-Silva et al., 2012; Akram et al., 2017; Zechmann, 2018; Bilska et al., 2019; Foyer et al., 2020). ROS produced by abiotic stress significantly influence the cell redox state. Changes in the cell redox state influence the ability of plants to respond to abiotic stress. Plants can respond rapidly and appropriately to such changes, to better adapt to various abiotic stresses, by monitoring their redox homeostasis (Potters et al., 2010; Sierla et al., 2013; You and Chan, 2015; Choudhury et al., 2017; Foyer, 2018; Waszczak et al., 2018; Farooq et al., 2019; Foyer et al., 2020). Based on its critical role in maintaining plant redox homeostasis, ASC can coordinate the actions of multiple signaling pathways in responses to abiotic stress, by modulating redox signaling (Pignocchi and Foyer, 2003; Akram et al., 2017; Bilska et al., 2019; Foyer et al., 2020). In this review, we will discuss the versatile roles of ASC as an antioxidant, cofactor, and regulator in plant adaptation to abiotic stress.

## As an Antioxidant, ASC Effectively Scavenges Ros and Enhances Abiotic Stress Tolerance

**Figure 1:**
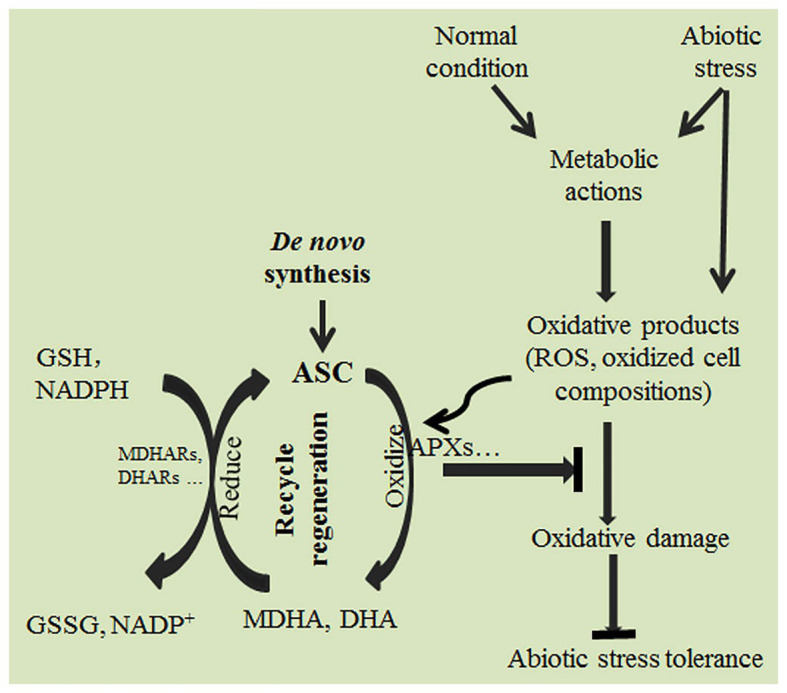
Ascorbate (ASC) effectively scavenges reactive oxygen species (ROS), eliminates oxidative stress, and enhances abiotic stress tolerance in plants *via de novo* ASC synthesis and recycling.

Under abiotic stress, plants produce ROS, such as hydrogen peroxide (H_2_O_2_), hypochlorous acid (HClO), ozone (O_3_), singlet oxygen (^1^O_2_), superoxide anion radicals (O_2_^−^), hydroxyl radicals (OH^−^), perhydroxyl radicals (HO_2_^•^), organic alkoxy (RO^•^), and organic peroxyl radicals (ROO^•^; Dumont and Rivoal, 2019; Dumanović et al., 2020). The accumulated ROS induced by abiotic stress are harmful and must be scavenged (Gill and Tuteja, 2010; You and Chan, 2015; Choudhury et al., 2017; Nadarajah, 2020). By regulating ASC *de novo* synthesis or recycle regeneration (Figure 1), plants can effectively scavenge many kinds of ROS directly or indirectly, maintaining cellular redox homeostasis (Noctor and Foyer, 1998; Smirnoff and Wheeler, 2000; Gallie, 2013; Akram et al., 2017; Bilska et al., 2019; Hasanuzzaman et al., 2019). ASC is thus critical in eliminating oxidative damage and enhancing abiotic stress tolerance. Photosynthesis can trigger a series of redox reactions that are accompanied by ROS production (Foyer, 2018; Khorobrykh et al., 2020). Excessive accumulation of radical and non-radical ROS in chloroplasts under light stress can damage the plant photosynthetic system. ASC effectively scavenges both types of ROS in chloroplasts, thus helping plants to sustain photosynthesis (Ivanov and Khorobrykh, 2003; Kramarenko et al., 2006; Triantaphylidès et al., 2008; Foyer, 2018; Khorobrykh et al., 2020). The radical ROS O_2_^−^ can be reduced to H_2_O_2_ by superoxide dismutase, and H_2_O_2_ can be then eliminated by ASC peroxidases (APXs) using ASC as an electron donor (Talla et al., 2011; Ivanov, 2014). The non-radical ROS ^1^O_2_ can be directly scavenged by ASC (Kramarenko et al., 2006). In addition, ^1^O_2_ can oxidize carotenoids and tocopherols; the oxidized tocopherols and carotenoids can then be reduced by ASC, indicating that ASC can scavenge non-radical ROS through directly and indirectly pathways (Veljovic-Jovanovic et al., 2001; Kramarenko et al., 2006; Jahns et al., 2009; Triantaphylidès and Havaux, 2009; Ivanov, 2014). In the *Arabidopsis* ASC synthesis mutant *vtc1*, the activity of the ASC synthesis key enzyme GDP-mannose phosphorylase is impaired, causing it to have only about 30% ASC of that in the wild type; it is therefore, much more prone to photooxidation and photoinhibition under light stress than the wild type (Conklin et al., 1996; Veljovic-Jovanovic et al., 2001; Ivanov, 2014). Similarly, the ASC synthesis mutant *vtc2*, which has only about 20% as much ASC as the wild type, exhibits decreased ROS scavenging ability and thus serious oxidative damage under high-intensity light (Müller-Moulé et al., 2004). These results suggest that ASC is crucial in protecting the photosynthetic system from oxidative damage (Noctor and Foyer, 1998; Talla et al., 2011; Ivanov, 2014; Khorobrykh et al., 2020).

Salinity, drought, and temperature stresses can cause ROS accumulation, resulting in severe oxidative damage (Cruz de Carvalho, 2008; Bhattacharjee, 2013; Choudhury et al., 2017). ROS scavenging is critical for plants to cope with these stresses (Sato et al., 2011; Lisko et al., 2013; Shigeoka and Maruta, 2014; Akram et al., 2017; Laxa et al., 2019; Broad et al., 2020a). Increasing ASC content by promoting ASC synthesis can enhance ROS scavenging ability and significantly improve plant stress tolerance (Hemavathi et al., 2000; Wang et al., 2011b, 2018; Zhang et al., 2012; Lisko et al., 2013; Ma et al., 2014; Ali et al., 2019; Broad et al., 2020a; Gaafar et al., 2020). In contrast, disruption of ASC *de novo* synthesis significantly reduces plant stress tolerance (Conklin et al., 1996; Noctor and Foyer, 1998; Huang et al., 2005; Qin et al., 2016; Wang et al., 2018).

Ascorbate regeneration *via* the ASC recycling pathway is also critical for plants to eliminate ROS damage and enhance abiotic stress tolerance (Sultana et al., 2012; Gallie, 2013; Caverzan et al., 2014; Sofo et al., 2015; Wang et al., 2017; Balfagón et al., 2018; Yeh et al., 2019; Broad et al., 2020b). APXs can effectively scavenge ROS with ASC, which is oxidized to monodehydroascorbate (MDHA); MDHA can then disproportionate to dehydroascorbate (DHA) and ASC (Smirnoff, 2000). ASC can be regenerated from MDHA and DHA by MDHA reductases (MDHARs) and DHA reductases (DHARs), respectively. Therefore, ASC regeneration can provide more ASC for ROS scavenging, and thus helps to maintain cell redox homeostasis and decrease oxidative damage under abiotic stress (Smirnoff, 2000; Chen et al., 2003; Li et al., 2010; Qin et al., 2011; Sultana et al., 2012; Gallie, 2013; Sofo et al., 2015; Lin et al., 2016; Broad et al., 2020b; Xiang et al., 2020). Drought can induce the expression of ASC recycling genes and thus, which enhances plant drought tolerance (Eltayeb et al., 2007; Gallie, 2013; Sofo et al., 2015; Diaz-Vivancos et al., 2016; Shan et al., 2018; Broad et al., 2020b). In tomato, the overexpression of *LeMDAR* improves the temperature stress tolerance of transgenic plants, whereas LeMDAR knockdown has the opposite effect (Li et al., 2010). In transgenic tobacco, the overexpression of DHAR in chloroplasts clearly enhances ROS scavenging capacity and improves plant cold tolerance (Le Martret et al., 2011). Similarly, the overexpression of DHAR enhances salt tolerance in rice, *Arabidopsis*, and tobacco (Kwon et al., 2003; Ushimaru et al., 2006; Le Martret et al., 2011; Sultana et al., 2012). On the contrary, deficiency of cytosolic DHAR impairs *Arabidopsis* abiotic stress tolerance (Yoshida et al., 2006). In summary, *de novo* synthesis and regeneration of ASC are both critical for plants to decrease ROS accumulation, eliminate oxidative damage, and enhance stress tolerance.

## As a Cofactor, ASC Participates in Abiotic Stress Responses by Regulating the Metabolism and Chemical Modification of Cell Components

As discussed above, ASC can directly scavenge ROS as the reducible substrate of antioxidant enzymes such as APXs (Chen et al., 2014). In addition, it can act as a cofactor of violaxanthin de-epoxidase (VDE) to indirectly eliminate ROS (Mu et al., 2002; Müller-Moulé et al., 2004; Jahns et al., 2009; De Tullio, 2012; Yang et al., 2017). VDE uses ASC as a substrate to reduce the xanthophyll pigment violaxanthin to zeaxanthin, which is able to dissipate excess excitation energy in the photosystem II light harvesting complex and protect the photosynthesis system from photooxidative stress (Jahns et al., 2009; Saga et al., 2010; Vidal-Meireles et al., 2020). Therefore, ASC deficiency inhibits zeaxanthin accumulation, resulting in serious photooxidative damage and impairing plants’ tolerance of abiotic stresses (Müller-Moulé et al., 2003, 2004; Plumb et al., 2018).

In addition to being a reducing substrate, ASC, also as a cofactor, is involved in the enzymatic reactions of several types of oxidases, such as 2-ODDs, glyceraldehyde-3-phosphate dehydrogenase, and cysteine oxidase (Jung and Wells, 1997; Shikita et al., 1999; Smirnoff, 2000; Arrigoni and De Tullio, 2002; Hedden and Thomas, 2012; Kawai et al., 2014). Among these, 2-ODDs are involved in various metabolic processes, such as protein hydroxylation and phytohormone synthesis and metabolism. These metabolic processes have important effects on plant abiotic stress responses, as well as on growth and development (Höller et al., 2015; Alegre et al., 2020; Broad et al., 2020b; Foyer et al., 2020). In humans, the role of ASC in scurvy is well understood. This disease is due to the lack of collagen, a protein critical for the structure of the extracellular matrix in humans. The collagen residues, hydroxyproline and hydroxylysine, are essential for its structural function. These residues are formed by peptidyl prolyl hydroxylases, which are 2-ODDs that need ASC as their cofactor (Myllyla et al., 1984; Myllyharju, 2003). In plants, there are several prolyl hydroxylases, such as prolyl 3-hydroxylase (P3H) and prolyl 4-hydroxylase (P4H), which can catalyze the hydroxyproline of polypeptides (Gorres and Raines, 2010). As in humans, plant prolyl hydroxylases also require ASC as their cofactor (Tiainen et al., 2005). Plant prolyl hydroxylases are involved in plant abiotic stress responses (Vlad et al., 2007; Asif et al., 2009; Iacopino and Licausi, 2020). They can be induced by hypoxia, and regulate the expression of hypoxia-responsive genes (Hieta and Myllyharju, 2002; Asif et al., 2009).

More importantly, several 2-ODDs, such as ACOs, NCEDs, and GA20 oxidases, are key enzymes for the synthesis of the phytohormones ethylene, ABA, and gibberellin (GA), respectively (Lange, 1994). These phytohormones are critical for plant stress responses, growth, and development (Wang et al., 2013; Colebrook et al., 2014; Sakata et al., 2014; Verma et al., 2016; Ciura and Kruk, 2018; Sahu and Kar, 2018). ASC participates in regulating plant abiotic stresses by regulating phytohormone synthesis (Sadak et al., 2013; Dinler et al., 2014; Terzi et al., 2015). For example, in wheat, ASC enhances salt tolerance by prompting the synthesis of GAs, indole acetic acid (IAA), zeatin, and brassinosteroids (BRs; Sadak et al., 2013). In tomato, higher amounts of reduced ASC contribute to induce ethylene synthesis, which further regulates fruit ripening and stress responses (Ioannidi et al., 2009). ASC can enhance maize drought tolerance by improving ABA synthesis, to decrease water loss and osmotic stress resistance (Terzi et al., 2015). Under heat stress, ASC can also enhance maize heat tolerance by decreasing ABA and IAA content, and increasing salicylic acid (SA) content (Dinler et al., 2014). Plants can coordinate the biosynthesis of different phytohormones to regulate growth and development processes, and thus adapt to internal and external conditions (Gururani et al., 2015; Verma et al., 2016; Ciura and Kruk, 2018; Sahu and Kar, 2018). GAs and ABA can be antagonistic in regulating plant growth and stress tolerance (Xu et al., 2015). ABA can suppress GA synthesis in tobacco seed embryos, thereby inhibiting germination. ASC can alter ABA and GA synthesis, thereby regulating plant growth and development processes and abiotic stress tolerance (Sadak et al., 2013; Dinler et al., 2014; Akram et al., 2017). Exogenous ASC can induce GA synthesis in tobacco seed embryos, and restore germination by counteracting the inhibitory effect of ABA on germination (Ye et al., 2012). In response to abiotic stresses, rice plants can increase ABA content and suppress GA synthesis to delay germination, *via* ASC (Liu et al., 2010). In *Arabidopsis*, ASC deficiency disrupts the synthesis of several hormones, such as ABA, GA, and SA, and alters flowering time and stress tolerance (Pastori et al., 2003; Barth et al., 2004, 2006; Huang et al., 2005). The ASC deficient mutant *vtc1* exhibits not only decreased stress tolerance, but also accelerated flowering, due to the enhanced SA synthesis under long-day condition. In contrast, under short-day condition, *vtc1* exhibits delayed flowering and more rapid senescence, *via* the inhibition of GA synthesis and the accumulation of ABA (Pastori et al., 2003; Barth et al., 2004, 2006). These results indicate that ASC may regulate plant growth and development, enabling plants to adapt to abiotic stresses, by coordinating phytohormone synthesis (Barth et al., 2006; Xu et al., 2015).

Additionally, ASC may participate in abiotic stress responses *via* epigenetic pathways. For humans, Chung et al. (2010) found that ASC causes widespread DNA demethylation in embryonic stem cells. In cultured animal cells, ASC can enhance 5-hydroxymethylcytosine (5hmC) generation as a cofactor for ten-eleven-translocation (TET) dioxygenase, a type of 2-ODD, which catalyzes the oxidation of 5-methylcytosine (5mC) into 5hmC (Dickson et al., 2013; Minor et al., 2013). The demethylation of genomic 5mC catalyzed by TET dioxygenase can lead to expression of the reprogramming gene (Young et al., 2015). Jumonji C (JMJC) histone demethylases, which are also 2-ODD enzyme, have an important role in histone demethylation. The activity of the JMJC enzymes KDM2A and KDM3A (JHMD2A) was correlated with the amount of ASC present (Tsukada et al., 2006). The histone demethylation regulated by ASC is important for regulating the chromatin state, somatic cell reprogramming, and gene expression (Wang et al., 2011a; Song et al., 2017; Zhang et al., 2019). Lu et al. (2008) showed that the function of histone demethylases, which are characterized by JMJC-type enzymes in plants, is similar to that in animals. In rice, histone demethylation has been shown to be critical for plant development and responses to abiotic stress (Chowrasia et al., 2018; Song et al., 2018). Although it has not been confirmed that TET catalyzes DNA demethylation in plants, genome-wide mapping of 5hmC in three rice cultivars revealed that 5hmC is present in significant amounts in the rice genome (Wang et al., 2015). Xue et al. (2019) showed that TET dioxygenase has an important role in the demethylation of the green alga genomic 5mC, indicating the TET-catalyzed DNA demethylation may occur in plants.

**Figure 2:**
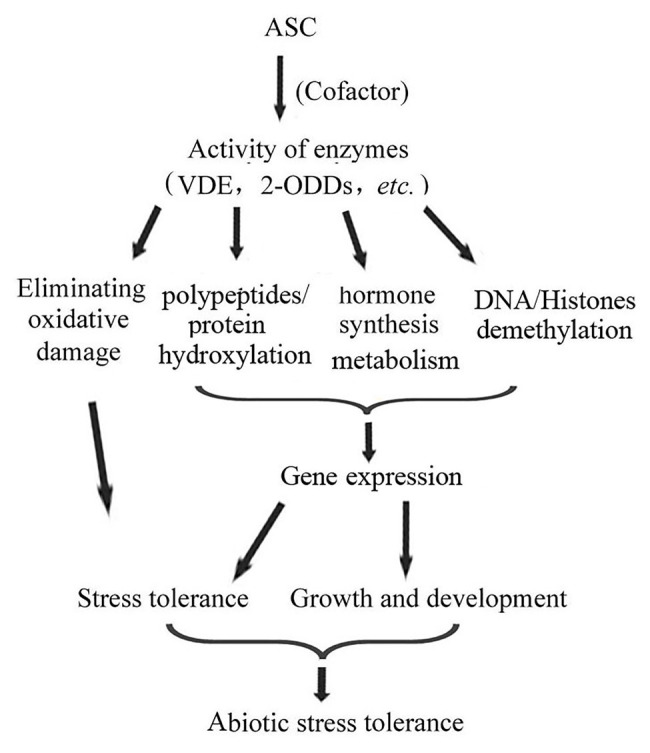
As a cofactor of various oxidases, ASC regulates plant growth and development and abiotic stress responses, enhancing plant abiotic stress tolerance, *via* multiple pathways.

Further, ASC can regulate gene expression by modifying transcription factor hydroxylation. In animals, P4H can catalyze the hydroxylation of the transcription factor hypoxia-inducible factor-1α (HIF-1α). The hydroxylation of two proline residues of HIF-1α leads to its ubiquitination and degradation; in contrast, when these two proline residues are non-hydroxylated, HIF-1α is transferred to the nucleus and activates the expression of downstream genes (Schofield and Ratcliffe, 2004; Frost et al., 2021). Similar to the function of P4H in animals, prolyl 4-hydroxylase (AtP4H) in *Arabidopsis* can hydroxylate proline-rich peptides, and enhance the transcription of hypoxia-responsive marker genes under hypoxia treatment (Asif et al., 2009). In summary, as an enzyme cofactor, ASC participates in abiotic stress responses by modifying plant cell composition, coordinating phytohormone biosynthesis, and regulating gene expression *via* epigenetic pathways (Figure 2).

## As a Regulator, ASC Manipulates Stress Signal Transduction and Coordinates Abiotic Stress Responses

In addition to acting as an antioxidant and cofactor, ASC also participates in plant abiotic stress responses as a regulator of plant cell signaling (De Gara et al., 2010; Choudhury et al., 2017; Waszczak et al., 2018; Bellini and De Tullio, 2019; Farooq et al., 2019; Alayafi, 2020). ASC greatly influences the actions of multiple signaling pathways, including the ROS and phytohormone signaling pathways. ASC thus integrates the actions of multiple signal pathways, and coordinates plant abiotic stress responses, by regulating the plant cell redox state (Pastori et al., 2003; Lima-Silva et al., 2012; Chen et al., 2014; Bellini and De Tullio, 2019; Bilska et al., 2019; Yu et al., 2019).

Reactive oxygen species play an important role in plant abiotic stress responses (Zhang and Guo, 2012; You and Chan, 2015; Nadarajah, 2020). In addition to causing oxidative damage, ROS can act as signaling molecules in activating the responses of plants to abiotic stresses (Foyer and Noctor, 2003; Finkel, 2011; Baxter et al., 2014; Schieber and Chandel, 2014; Choudhury et al., 2017; Hasanuzzaman et al., 2020). Therefore, the regulation of ROS homeostasis is critical for plants to adapt to abiotic stresses (Suzuki and Katano, 2018; Nadarajah, 2020). Plants can effectively manipulate cell ROS homeostasis under abiotic stress by regulating *de novo* ASC synthesis, or *via* ASC recycling regeneration (Wang et al., 2010; Gallie, 2013; Sofo et al., 2015; Noshi et al., 2017; Bilska et al., 2019; Broad et al., 2020b; Guo et al., 2020). In addition to scavenging the accumulated ROS to eliminate oxidative damage, ASC can control ROS signal transduction by regulating ROS homeostasis (Chen and Gallie, 2004; Pignocchi et al., 2006; De Tullio et al., 2013; Cobley et al., 2015; Bellini and De Tullio, 2019; Foyer et al., 2020). Unlike the enzymes involved in *de novo* ASC synthesis, which mainly serve to eliminate oxidative damage and maintain normal physiological and biochemical activation in plants, the enzymes that perform ASC metabolism and regeneration are also critical in regulating cell redox signals (Fotopoulos et al., 2006; Bellini and De Tullio, 2019).

ASC peroxidases, which are critical enzymes in ASC metabolism, play an important role in both ROS scavenging and manipulating the activity of various cell signaling pathways. APXs are distributed in various organs and are involved in sustaining cellular redox homeostasis (Bonifacio et al., 2011; Chen et al., 2014; Maruta et al., 2016; Yu et al., 2019). In *Arabidopsis*, the deficiency of APX6 activity decreases the content of reduced ASC in the seed, and promotes DHA accumulation, which disrupts cell redox homeostasis and further affects the action of ROS, ABA, and auxin signaling pathways (Chen et al., 2014). Similarly, silencing of *Arabidopsis* thylakoid membrane-bound APX disturbs the expression of downstream genes in the H_2_O_2_ signaling pathway of chloroplasts (Maruta et al., 2012, 2016).

Ascorbate oxidases (AOs), which also oxidize ASC to MDHA, are involved in cell signal transduction (Szarka et al., 2004; Pignocchi et al., 2006; Li et al., 2007, 2017; De Tullio et al., 2013; Bellini and De Tullio, 2019; Pan et al., 2019). Unlike APXs, which are located in various parts of plant cells, AOs are mostly located in the cell wall, and oxidize apoplastic ASC (Pignocchi et al., 2006; De Tullio et al., 2013). In the apoplast, MDHA arising from ASC oxidation by AOs can be converted to DHA and rapidly transported into the cytoplasm, where it can then be recycled into ASC by DHARs. In contrast, the reduced ASC in the cytoplasm can be transferred to the apoplast, resulting in ASC exchange between the apoplast and cytoplasm. This DHA-ASC exchange is critical for maintaining redox homeostasis in both the apoplast and cytoplasm (Sanmartin et al., 2003; Fotopoulos et al., 2006; De Tullio et al., 2013; Gallie, 2013; Pan et al., 2019; Foyer et al., 2020). Under abiotic stress, the DHA flux from apoplastic oxidization of ASC increases rapidly and disrupts the balance of the redox state in the apoplast and cytoplasm; this acts as a signal to initiate a response to adverse environmental conditions (Horemans et al., 2000; Sanmartin et al., 2007). The expression of AOs can be induced by abiotic stresses, and their expression level has an important effect on plant abiotic stress tolerance (Esaka et al., 1992; Li et al., 2017; Pan et al., 2019). Another difference between APXs and AOs is that APXs oxidize ASC by using H_2_O_2_, whereas AOs consume ASC by using O_2_. The oxidized ASC (DHA) can further produce various metabolites by AOs under plant apoplastic conditions. Among these products, some may delay APXs action and inhibit ROS scavenging. Moreover, some products, such as 2,3-diketogulonate, produce H_2_O_2_ by AOs or non-enzymatical pathway (Parsons and Fry, 2012; Kärkönen et al., 2017; Dewhirst and Fry, 2018; Smirnoff, 2018; Dewhirst et al., 2020). Therefore, unlike APXs, which use ASC to scavenge ROS and eliminate oxidative damage, AOs consume ASC to accelerate the accumulation of ROS in apoplast. Thus, the overexpression of AOs decreases abiotic stress tolerance, due to the enhanced activities of AOs both disrupting the normal stress signal flux from the apoplast to the cytoplasm and increasing the oxidative damage from the accumulated ROS (Fotopoulos et al., 2006; Garchery et al., 2013). The role of AOs in regulating the activity of cell signaling pathways has been demonstrated by the discovery of close links between AOs and ROS signaling in the stress response, growth, and development of cotton (Li et al., 2007, 2017; Pan et al., 2019; Yu et al., 2019). The expression of the cotton AO genes *GhAO1* and *GhAO1A* can modulate apoplastic ROS homeostasis and hormone signaling, which affects not only plant stress tolerance, but also cell elongation and leaf senescence, respectively (Li et al., 2007, 2017; Pan et al., 2019).

Ascorbate regeneration is also involved in regulating plant signaling. DHARs are responsible for the regeneration of ASC from DHA, which plays an important role in transmitting abiotic stress signals (Chen and Gallie, 2004; Rahantaniaina et al., 2017). For example, DHAR-overexpression in the stoma of tobacco promotes the production of reduced ASC and decreases plant drought tolerance; because it blocks guard cells from responding to ABA and H_2_O_2_ signaling, and keeps stomatal opening and increases water loss (Chen and Gallie, 2004). In contrast, suppressing DHAR expression in stoma promotes H_2_O_2_ accumulation, which triggers ABA and H_2_O_2_ signaling, promoting stomatal closure and decreasing water loss; suppressing DHAR activity in the stoma can therefore enhance drought tolerance (Chen and Gallie, 2004; Gallie, 2013).

**Figure 3:**
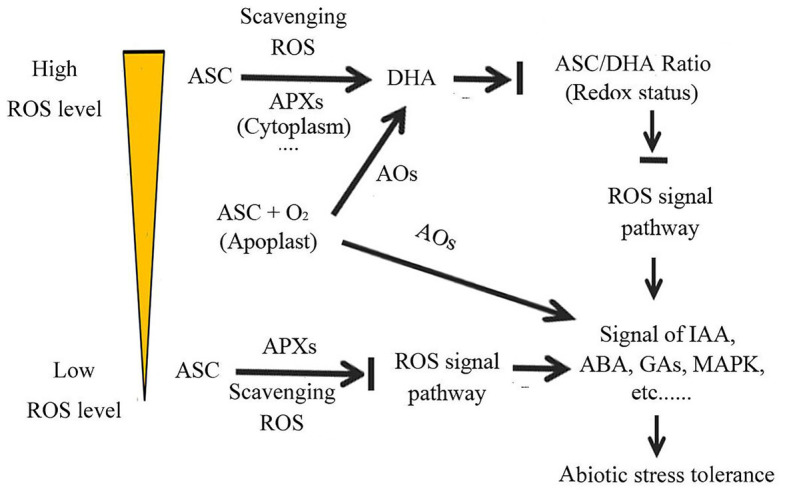
Ascorbate regulates the cellular redox state, thereby coordinating various signaling pathways, to enhance plant adaptability to abiotic stress.

The ratio of ASC to DHA (ASC/DHA ratio) plays an important role in transmitting plant cellular redox signal (Pignocchi and Foyer, 2003; Chen and Gallie, 2004; De Tullio et al., 2013; Sierla et al., 2013; Cobley et al., 2015). ROS functions are closely related to their concentrations. At low concentrations, they act as signaling molecules to activate the plant stress response system to cope with adverse condition, whereas at high concentrations, they cause oxidative damage to plants (Schieber and Chandel, 2014; Choudhury et al., 2017). According to the ROS content, plants take different measures to deal with ROS (Figure 3). When ROS are present at low levels, and act as signaling molecules, plants can utilize ROS to transmit the cellular redox state signal, by using ASC to negatively regulate the ROS signaling. In contrast, when ROS are present at high levels, plants use ASC to scavenge ROS, to avoid the oxidative damage caused by ROS, and use the ASC-DHA redox couple to transmit the cellular redox state signal (Noctor and Foyer, 1998; Smirnoff and Wheeler, 2000; Potters et al., 2010; Schieber and Chandel, 2014; Choudhury et al., 2017). The ASC/DHA ratio is therefore central for plants to transfer abiotic stress signals caused by adverse environmental conditions (de Pinto et al., 1999; Foyer and Noctor, 2003; Eastmond, 2007; Anjum et al., 2014; Foyer et al., 2020). The ASC/DHA ratio can affect the action of auxin- and calcium-ion signaling, and further affect the abiotic stress response (Pignocchi and Foyer, 2003; Yamamoto et al., 2005; Fotopoulos et al., 2006; Pignocchi et al., 2006; Sanmartin et al., 2007). The low ASC/DHA ratio in the apoplast inhibits the response of plants to auxin, which is due to the inhibition of auxin signal transduction across the plasma membrane (Barbier-Brygoo et al., 1989; Pignocchi and Foyer, 2003; Pignocchi et al., 2006). DHAR deficiency decreases the ASC/DHA ratio and impairs SA signaling in *Arabidopsis* (Rahantaniaina et al., 2017). Under copper stress, impaired DHAR expression substantially decreases the ASC/DHA ratio and blocks the MAPK signaling pathway, in order to cope with copper stress (Rodríguez-Rojas et al., 2019). Therefore, a high ASC/DHA ratio can represent a strong ability to cope with abiotic stress. For instance, in *Arabidopsis*, impaired MDAR4 function decreased the AsA/DHA ratio without affecting the ASC content, but substantially impaired plant oxidative stress tolerance (Eastmond, 2007). In tobacco, SA can elevate DHAR activity and increase the ASC/DHA ratio, which can prompt the scavenging of ROS accumulated by abiotic stress, and thus enhance plant salt tolerance (Yan et al., 2018).

The question arises of how the roles of ASC in scavenging ROS and regulating ROS signal under abiotic stresses can be distinguished. Before answering this question, it is necessary to analyze the role of ASC in plant abiotic stress responses. Studies have shown that ACS plays a double role in the response to abiotic stress, and in growth and development (De Tullio, 2012). As Bellini and De Tullio (2019) discussed for the role of ASC under ozone treatment, there is no evidence that ASC content or the activity of ASC regeneration enzymes is related to the antioxidant capacity of plants. Although high ASC content or strong ASC regeneration capacity is considered to improve plant abiotic stress tolerance, there are many cases in which enhancing ASC content or regenerating enzyme activity decreases plants abiotic stress tolerance. The importance of ASC in transmitting abiotic stress-related signals may well explain why ASC enhances ROS scavenging ability, but results in decreased plant abiotic stress tolerance (De Tullio et al., 2013). ROS cause oxidative damage, but also play an important role in activating the responses of plants to stresses (Foyer and Noctor, 2003; Finkel, 2011; Baxter et al., 2014). “Hormesis” refers to a phenomenon in which low levels of the stressor induce optimal plant growth, whereas higher levels of the same stressor damage plants (Oliveira et al., 2018). Enhanced ROS scavenging ability disrupts the generation of ROS signals, which are responsible for the transmission of abiotic stress signals under low ROS levels. Inhibition of the ROS signaling pathway severely delays or impairs plant “hormesis” effect, and decreases plants abiotic stress tolerane (Calabrese and Mattson, 2017; Agathokleous et al., 2019).

Ascorbate may induce a biphasic response in plants under abiotic stresses. In ABA- and ROS-mediated stress responses, ASC exhibits this biphasic response to environmental stress (De Tullio, 2012). ABA may play a dual role in regulating plant ROS levels under abiotic stress. In the early stage of stress response, ABA promotes ROS production and activates ROS signaling, thereby helping plants to respond rapidly to adverse environmental conditions (Kwak et al., 2003; Liu et al., 2010; Sahu and Kar, 2018). In contrast, during the late stage of stress adaptation, ABA activates the *de novo* synthesis and regeneration of ASC to scavenge the accumulated ROS and thus eliminate oxidative damage, to enhance plant abiotic stress tolerance (Zhang et al., 2020). This may explain why ABA inhibits ASC synthesis and accelerates ROS accumulation in some circumstances (Jiang and Zhang, 2002; Kwak et al., 2003; Yu et al., 2019), but induces ASC synthesis and ROS scavenging in others (Jiang and Zhang, 2002; Zhang et al., 2020). Therefore, we hypothesize that ASC plays a more important role in regulating plant stress signal transduction at low cellular oxidation levels, and a more critical role in scavenging ROS at high cellular oxidation levels (Figure 3). In fact, in some cases, ASC may play different roles at the same time. In the apoplast, ASC does not protect cell wall components from oxidative damage, instead being involved in external signal transduction. ASC can act as a cofactor to keep iron (Fe) in the Fe^2+^ state and protect 2-ODDs from oxidative damage (Myllyla et al., 1984; Kivirikko and Pihlajaniemi, 1998; Wu et al., 2000; Hoffart et al., 2006). In summary, ASC can integrate the actions of multiple signaling pathways by modulating the cell redox state; this integration is critical in abiotic stress responses. The role of ASC in cell signal transduction is related to its cellular location, the ROS concentration, and the stage of the plant stress response (Figure 3).

## Discussion and Conclusion

Antioxidation is traditionally considered as the primary role of ASC in plant responses to abiotic stresses (Noctor and Foyer, 1998). However, ASC has been shown to act as an enzyme cofactor or regulator of cell signaling, coordinating phytohormone synthesis, and the actions of various signaling pathways, thereby adjusting plant growth and development processes and stress responses in adaptation to ever-changing internal and external conditions (Arrigoni and De Tullio, 2002; De Tullio, 2012; Bellini and De Tullio, 2019; Foyer et al., 2020).

Ascorbate plays multiple roles in abiotic stress responses. First, as an antioxidant, ASC directly or indirectly scavenges the ROS produced by abiotic stress, to eliminate oxidative damage and enhance plant abiotic stress tolerance (Figure 1). Second, as a cofactor, it regulates the synthesis and metabolism of various cell components, including phytohormones, thereby profoundly influencing the integration of plant stress responses and growth and development processes (Figure 2). Third, it can regulate the activities of various signaling pathways (Figure 3). In responding to abiotic stress, plants effectively coordinate the actions of various signaling pathways, such as hormone, ROS, and MAPK signaling pathways, by quickly regulating cellular redox signaling *via* ASC (by altering the ASC/DHA ratio), thereby rapidly responding and adapting to abiotic stresses (Pastori et al., 2003; Ye et al., 2012; Smirnoff, 2018). Notably, the roles of ASC in plant responses to abiotic stress should be analyzed according to its specific cellular location, the cellular ROS content, and the stage of the stress response (Zechmann, 2018).

Ascorbate is a complex and multifaceted cellular compound, with many functions that remain to be elucidated in plants. Although its central role in regulating plant redox signals has been well described, the mechanisms by which it regulates the cell redox status to coordinate the balance between cell redox signaling and ROS scavenging remain unknown. The fluctuation of the ASC/DHA ratio has important effects on growth and development processes and plant adaptation to abiotic stresses; nonetheless, it remains unclear what regulatory mechanisms and pathways are involved in maintaining the dynamic balance of ASC/DHA ratio (Yamamoto et al., 2005; Tripathi et al., 2012; Foyer et al., 2020). In addition, in animals, ASC participates in regulating gene expression, is involved in DNA and histone demethylation, and alters the cell cycle *via* epigenetic pathways (Blaschke et al., 2013; Xue et al., 2019). However, its role in DNA demethylation in plants remains unclear. Further studies on the effects and roles of ASC in the plant epigenome will expand the understanding of this important micronutrient.

## Author Contributions

MX, BpZ, FZ, HZ, and YW: wrote introduction, discussion, and ASC as a cofactor. ZL and JW: wrote ascorbate as an antioxidant. LZ, MX, and FZ: wrote ASC as a regulator. ZL, YW, BoZ, FZ, and LZ: original draft preparation. MX, ZL, LZ, JW, and ZZ: writing review and editing. ZZ: supervision and funding acquisition. All authors contributed to the article and approved the submitted version.

### Conflict of Interest

The authors declare that the research was conducted in the absence of any commercial or financial relationships that could be construed as a potential conflict of interest.
